# Additive Effect in the Induction of Kidney Tumours in Rats Treated with Dimethylnitrosamine and Ethylmethanesulphonate*

**DOI:** 10.1038/bjc.1974.6

**Published:** 1974-01

**Authors:** R. Montesano, U. Mohr, P. N. Magee, J. Hilfrich, H. Haas

## Abstract

**Images:**


*Br. J. Cancer* (1974) **29**, 50

### ADDITIVE EFFECT IN THE INDUCTION OF KIDNEY TUMOURS IN RATS TREATED WITH DIMETHYLNITROSAMINE AND ETHYLMETHANESULPHONATE*

R. MONTESANO, U. MOHR, P. N. MAGEE, J. HILFRICH AND H. HAAS

*From the International Agency for Research on Cancer, Lyon, France; Section of Experimental Pathology, Medizinische Hochschule, Hannover, Federal Republic of Germany and Courtauld Institute of Biochemistry, Middlesex Hospital Medical School, London, England*

Received 3 September 1973. Accepted 17 October 1973

**Summary.—Wistar rats were treated with a single dose of 30 mg/kg of DMN or with single doses of 100, 200 or 300 mg/kg of EMS. Tumours of the kidney developed in a few animals receiving EMS and in 33% of the male and 63% of the female rats treated with DMN alone. In the animals receiving DMN and, 8 hours later, a single dose of 100, 200 or 300 mg/kg of EMS, an additive effect was observed in the induction of kidney tumours. This additive effect was more pronounced in female than in male rats. Morphologically, the tumours were of epithelial and mesenchymal type with a preponderance of the former type. The significance of alkylation of the nucleic acids of the kidney observed with these two compounds is discussed in relation to the present findings.**

A SINGLE dose of dimethylnitrosamine (DMN), which is completely metabolized within 5–6 hours, is sufficient to produce a significant incidence of kidney tumours in the rat (Magee and Barnes, 1959). Ethylmethanesulphonate (EMS) administered in 3 large doses resulted in an increase of lung adenomata and induced epithelial cell tumours of the kidney in mice (Alexander and Connell, 1963) and tumours of the kidney in rats (Swann and Magee, 1969). Biochemical studies showed that administration of a single dose of DMN or EMS to rats results in the alkylation of some cellular constituents of the kidney, following metabolic activation in the case of DMN and spontaneous breaking down in the case of EMS (Swann and Magee, 1968, 1971).

Although the significance of alkylation of cellular macromolecules in initiating a carcinogenic process is not clear, it appears that it is important for the carcinogenic action of DMN and EMS. The induction of kidney tumours after treatment with a single dose of these two alkylating agents is a promising model which allows a study of the correlation between biological response and biochemical interactions with cellular components. The following long-term studies were carried out in order to examine a possible additive effect in the induction of kidney tumours, which might be the result of the total level of alkylation of cellular components after combined treatment with DMN and EMS.

### MATERIALS AND METHODS

The materials used were dimethylnitrosamine (DMN), (obtained from Dr F. Krüger), ethylmethanesulphonate (EMS), (T. Schuchardt GmbH Co., Munich, Federal Republic of Germany), and 0·9% sodium chloride. Groups of Wistar rats of both sexes, 9–10 weeks old, from the colony of the Zentral Institut für Versuchstiersucht der Deutschen Forschungsgemeinschaft, Hannover, were

* Presented in part at The Fifth Quadrennial International Conference on Cancer, Perugia, Italy, June 1973.

employed. All animals were observed and weighed once weekly; they were killed 110 weeks after the treatment, by which time all the animals of Group 7 had died spontaneously. Except for a few animals lost through cannibalism, all were autopsied. All the organs, including the central nervous system, were examined macroscopically. Eight graded histological sections were made for each kidney and other pertinent tissues were processed for histological examination.

Groups 1, 2 and 3 received a single i.p. injection of 100, 200 and 300 mg/kg respectively of EMS in 1·0 ml of 0·9% sodium chloride. The solution of EMS was freshly prepared before treatment. Group 4 received a single i.p. injection of 30 mg/kg of DMN in 1·0 ml of 0·9% sodium chloride. The other groups (Groups 5, 6 and 7) received a single i.p. injection of DMN as in Group 4 and 8 hours later an i.p. injection of 100, 200 or 300 mg/kg of EMS respectively.

A control group received a single i.p. injection of 1·0 ml of 0·9% sodium chloride. The number of rats in each group is given in Table I.

TABLE I.—*Tumour Induction in Rats following Treatment with DMN and/or EMS*

| Groups | Initial no. of animals | Effective no. of animals[a] | Total tumour bearing animals | No. of animals with tumours of: Kidney | Nasal cavities | Mammary glands | Lung | Other organs |
|---|---|---|---|---|---|---|---|---|
| Control | 20 ♂ | 20 | 4 | — | — | — | — | 4[b] |
| | 20 ♀ | 20 | 16 | — | — | 9 | — | 10[c] |
| 1 | 20 ♂ | 20 | 5 | 2 | — | — | — | 3[d] |
| EMS 100 mg | 20 ♀ | 18 | 12 | 1 | — | 9 | — | 7[e] |
| 2 | 20 ♂ | 20 | 8 | — | — | 1 | — | 7[f] |
| EMS 200 mg | 20 ♀ | 20 | 14 | — | — | 13 | — | 5[g] |
| 3 | 20 ♂ | 20 | 9 | 2 | — | 1 | 1 | 7[h] |
| EMS 300 mg | 20 ♀ | 20 | 13 | — | — | 8 | — | 9[i] |
| 4 | 19 ♂ | 15 | 10 | 5 | 3 | 1 | 2 | 5[j] |
| DMN 30 mg | 21 ♀ | 19 | 18 | 12 | 5 | 6 | — | 3[k] |
| 5 | 20 ♂ | 18 | 13 | 9 | 4 | 1 | 2 | 7[l] |
| DMN + EMS-100 | 20 ♀ | 18 | 12 | 11 | 2 | 4 | 1 | 6[m] |
| 6 | 25 ♂ | 16 | 12 | 8 | 2 | — | 4 | 4[n] |
| DMN + EMS-200 | 20 ♀ | 17 | 15 | 10 | — | 6 | 1 | 8[o] |
| 7 | 35 ♂ | 14 | 11 | 9 | 2 | 2 | 2 | 7[p] |
| DMN + EMS-300 | 40 ♀ | 21 | 20 | 15 | 6 | 2 | 2 | 9[q] |

[a] Survivors at time of first tumour (11 weeks), less animals lost through cannibalism.

[b] 1 malignant lymphoma; 1 adrenal gland adenoma; 1 hepatoma; 1 tumour of the testis.

[c] 6 malignant lymphomata; 1 adrenal gland adenoma; 1 insuloma; 2 uterus carcinomata; 1 lipoma; 1 forestomach papilloma; 1 Zymbal gland squamous cell carcinoma.

[d] 1 malignant lymphoma; 1 adrenal gland adenoma; 1 fibrosarcoma of the heart; 1 intestinal adenocarcinoma.

[e] 2 malignant lymphomata; 2 uterus polyps; 1 squamous cell carcinoma of the vagina; 1 s.c. fibrosarcoma; 1 forestomach papilloma.

[f] 3 malignant lymphomata; 1 phaeochromocytoma; 1 lipoma; 1 hepatocellular carcinoma; 1 thyroid carcinoma; 1 squamous cell carcinoma of the palate.

[g] 1 malignant lymphoma; 1 adrenal gland adenoma; 1 fibrosarcoma of the heart; 1 hepatoma; 1 forestomach papilloma.

[h] 4 adrenal gland adenomata; 1 fibrosarcoma of the heart; 1 squamous cell carcinoma of the palate; 1 carcinoma of the sebaceous glands; 2 papillomata of the tongue; 1 thyroid carcinoma.

[i] 2 malignant lymphomata; 5 fibrosarcomata of the heart; 1 granulosa cell carcinoma; 1 polyp of the cervix; 1 luteoma; 1 bladder papilloma; 1 uterus haemangioma.

[j] 3 malignant lymphomata; 1 fibrosarcoma of the heart; 2 adrenal gland adenomata; 1 s.c. fibrosarcoma.

[k] 1 malignant lymphoma; 1 hepatoma; 1 intestinal haemangioma.

[l] 5 adrenal gland adenomata; 2 fibrosarcomata of the heart.

[m] 1 malignant lymphoma; 1 adrenal gland adenoma; 1 thyroid carcinoma; 1 adenocarcinoma of the ovary; 1 oesophageal papilloma; 1 insuloma.

[n] 2 adrenal gland adenomata; 1 fibrosarcoma of the heart; 1 thyroid carcinoma; 1 papilloma of the tongue.

[o] 4 malignant lymphomata; 3 adrenal gland adenomata; 1 squamous cell carcinoma of the palate; 1 intestinal sarcoma; 1 hepatoma; 1 osteosarcoma.

[p] 3 malignant lymphomata; 2 adrenal gland adenomata; 1 fibrosarcoma of the heart; 1 hepatocellular carcinoma; 1 hepatoma; 1 papilloma of the tongue.

[q] 5 malignant lymphomata; 2 adrenal gland adenomata; 1 fibrosarcoma of the heart; 1 hepatocellular carcinoma; 1 forestomach papilloma; 1 luteoma; 1 papilloma of the tongue.

### RESULTS

During the first 10 weeks, no difference in survival rates was observed between control and treated groups, except for Groups 6 and 7 receiving the highest dose of EMS in combination with DMN, where a high mortality occurred within the first week due to toxicity of the combined treatment. Subsequently, a similar mortality occurred among the rats of all groups up to 60 weeks. From this time, a greater mortality was noticed in Groups 6 and 7, due to the appearance of tumours. Since all the animals of Group 7 were dead by 110 weeks, we decided to sacrifice the animals surviving among the other groups as well at this time.

In the control groups, none of the animals developed kidney tumours. Tumours were found in 4 of the males; they were a malignant lymphoma at 11 weeks, one adrenal cortical adenoma at 102 weeks, one hepatoma and a testicular tumour at 110 weeks. Out of 20 females, 9 developed mammary gland tumours and 6 had malignant lymphomata between 85 and 110 weeks. A few additional tumours scattered in various organs, developed after the 110 weeks (Table I).

### *Treatment with DMN or EMS alone*

The tumour incidences for all groups are given in Table I. In Groups 1 and 3, receiving a single dose of 100 or 300 mg/kg of EMS, 5 out of 78 animals of both sexes developed kidney tumours at an average latent period ranging from 90 to 95 weeks. No tumours of the kidney were observed in Group 2. One rat of Group 3 showed a lung tumour at 97 weeks. The incidence of mammary gland tumours, malignant lymphomata as well as other tumours was similar to that observed in the control group.

In Group 4, receiving a single dose of 30 mg/kg body weight of DMN, tumours of the kidney developed in 33% of the males and in 63% of the females, the average time of appearance being 85–90 weeks. In addition, 8 rats developed tumours of the nasal cavities and 2 out of 34 tumours of the lung. Tumours of other sites showed similar incidences to those in the control group.

### *Combined Treatment with DMN and EMS*

In Groups 5, 6 and 7 the final incidences of kidney tumours were 50, 50 and 64% in the males and 61, 59 and 71% in the females respectively (Table I).

These data showed an increased incidence of kidney tumours in the males compared with the 33% incidence observed in the males of Group 4 receiving DMN alone. The females of Group 5 and 6 showed a similar final incidence of kidney tumours to that of Group 4 (63%), whereas the highest tumour yield (71%) was observed in Group 7, receiving the highest dose of EMS in combination with DMN.

Comparison of the percentage of tumour incidences based on the initial number of animals can be misleading if deaths due to causes other than the observed tumours occur at different rates in the various experimental groups. For this reason, the progression with time of the cumulative probability for observing a kidney tumour at death has been calculated by the actuarial method described by Kaplan and Meier (1958). The actuarial probabilities for observation of kidney tumours at death in animals of Groups 1, 3, 4, 5, 6 and 7 have been plotted on Fig. 1, 2 and 3. When males and females (Fig. 1) are examined together, a considerable shortening of the latent period for these tumours was observed in the rats receiving the combined treatments when compared with rats receiving DMN or EMS alone. However, some differences appear when males and females are considered separately. In the females (Fig. 2), all the rats receiving the combined treatment show a shortening of the latent period with no variation among these 3 groups, whereas in the males (Fig. 3) an earlier appearance of kidney tumours was confined to Group 7. Statistically,

FIG. 1.—Probability for the observation of a kidney tumour in male and female rats at death, calculated according to Kaplan and Meier (1958). ○———○, Group 1; ○- - - - ○, Group 3; ●———●, Group 4; ×———×, Group 5; × - - - - ×, Group 6; +—·—·+, Group 7. No kidney tumours were observed in Group 2.

FIG. 2.—Probability for the observation of a kidney tumour in female rats at death, calculated according to Kaplan and Meier (1958). ●———●, Group 4; ×———×, Group 5; × - - - - ×, Group 6; +—·—·+, Group 7. One kidney tumour was observed in a rat of Group 1 at 89 weeks.

FIG. 3.—Probability for the observation of a kidney tumour in male rats at death, calculated according to Kaplan and Meier (1958). ●———●, Group 4; ×———×, Group 5; ×- - - -×, Group 6; +—·—·+, Group 7. Two kidney tumours were observed in rats of Group 1 and Group 3 respectively, dying between 88 and 96 weeks.

the greater incidence of kidney tumours observed in female and male rats of Group 7 was highly significant when compared with the rats of Group 4, as shown in Table III, where the instantaneous tumour mortality rates and the associated $\chi^2$ values relative to the DMN alone treated rats for the 3 groups receiving the combined DMN plus EMS treatment were calculated according to Mantel (1966). The rats of Groups 5 and 6 showed a tendency to a greater tumour mortality but these results failed to obtain a statistical significance with the exception of borderline values for the females of Group 5.

TABLE II.—*Multiplicity of Tumours Induced in Rats by DMN and/or EMS*

| Group | | Total tumour bearing animals | Total no. of tumours | Ratio |
|---|---|---|---|---|
| Control | ♂ | 4 | 4 | 1·0 |
| | ♀ | 16 | 23 | 1·43 |
| 1 | ♂ | 5 | 6 | 1·20 |
| | ♀ | 12 | 17 | 1·41 |
| 2 | ♂ | 8 | 9 | 1·12 |
| | ♀ | 14 | 19 | 1·35 |
| 3 | ♂ | 9 | 15 | 1·66 |
| | ♀ | 13 | 22 | 1·69 |
| 4 | ♂ | 10 | 19 | 1·90 |
| | ♀ | 18 | 47 | 2·61 |
| 5 | ♂ | 13 | 33 | 2·53 |
| | ♀ | 12 | 34 | 2·83 |
| 6 | ♂ | 12 | 32 | 2·66 |
| | ♀ | 15 | 41 | 2·73 |
| 7 | ♂ | 11 | 40 | 3·63 |
| | ♀ | 20 | 64 | 3·20 |

FIG. 4.—Large mesenchymal tumour of the kidney. Group 5. ×1·5.

FIG. 5.—Bilateral and multiple adenocarcinomata of the kidney. Group 7. ×1·5.

FIG. 6.—Part of a kidney adenoma demonstrating expansive growth. Group 5. H. & E. ×120.

FIG. 7.—Mesenchymal tumour of the kidney. Group 4. H. & F. ×300.

TABLE III.—*Instantaneous Kidney Tumour Mortality Rates Relative to DMN Alone (RR), and Associated $\chi^2$ Values, for the Three Groups treated with DMN plus EMS, by Sex (Mantel,* 1966)

| Sex | DMN + 100 mg EMS | | | DMN + 200 mg EMS | | | DMN + 200 mg EMS | | |
|---|---|---|---|---|---|---|---|---|---|
| | RR | $\chi^2$ | $P$ | RR | $\chi^2$ | $P$ | RR | $\chi^2$ | $P$ |
| ♀ + ♂ | 0·4381 | 4·06 | <0·05 | 0·5912 | 1·6461 | ~0·20 | 0·2448 | 15·9863 | <0·0001 |
| ♀ | 0·3201 | 3·8668 | <0·05 | 0·4178 | 2·2888 | ~0·13 | 0·2885 | 7·3636 | <0·01 |
| ♂ | 0·6069 | 0·267 | ~0·60 | 0·6114 | 0·3156 | ~0·57 | 0·1767 | 6·6659 | <0·01 |

Among the other types of tumours, no differences were observed between these groups and the DMN-alone treated group, with the possible exception of lung tumours which occur in a slightly higher number.

Another indication of the additive effect of EMS and DMN in their carcinogenic action is given by the multiplicity of tumours induced. As shown in Table II, the ratio of the total number of tumours over the total tumour bearing animals markedly increases in the groups treated with DMN plus EMS.

### *Morphology*

The morphological aspects of the kidney tumours reported here were analogous to those previously described by various authors (Magee and Barnes, 1962; Hard and Butler, 1970; Riopelle and Jasmin, 1969). They were of 2 histological types—epithelial (Fig. 6) and mesenchymal (Fig. 7), which developed as multiple growths localized in the cortical region (Fig. 5). The mesenchymal tumours appeared as large growths with a tendency to necrosis and haemorrhage (Fig. 4). Occasionally metastases were observed in the lung. A preponderance of adenomata and/or adenocarcinomata over mesenchymal tumours was observed in all the groups. In the EMS-alone treated rats (Groups 1 and 3) bearing a total of 5 kidney tumours, 3 were epithelial and 2 mesenchymal. In the other groups, 90% of the kidney tumours were of the epithelial type. No differences were detected among females and males on the tumour type.

The tumours of the nasal cavities were of various types, such as squamous cell papillomata and carcinomata, adenomata, adenocarcinomata and undifferentiated carcinomata. The lung tumours were 8 adenomata, 4 adenocarcinomata and 3 squamous cell carcinomata.

### DISCUSSION

The present studies show an additive effect in the induction of kidney tumours in rats following combined treatment with single doses of EMS and DMN. In addition, the carcinogenicity of EMS has been confirmed.

Previous studies (Swann and Magee, 1969) in female Wistar rats showed that 3 doses of 275 mg/kg of EMS administered over a period of a week were necessary to induce mesenchymal kidney tumours in 50% of the rats, whereas after a single dose of 350 mg/kg, a brain tumour was observed but no kidney tumours. Our results show that a single dose of 100 or 300 mg/kg is sufficient to induce a low incidence of kidney tumours in these rats after a latent period of 90–95 weeks. The fact that no kidney tumours were observed in the studies of Hrushesky, Sampson and Murphy (1972) might be attributed to the early sacrifice of the animals. The rats, treated with DMN alone (Group 4), showed an incidence of 33% of kidney tumours in the males which is comparable with previous data (McLean and Magee, 1970; Schmidt and Murphy, 1966). The final yield of kidney tumours in females reached an incidence of 63%. A high susceptibility of the females has been previously reported in Wistar and Sprague–Dawley rats (Magee and Barnes, 1962; Riopelle and Jasmin, 1969).

The additive effect between EMS and DMN in the induction of kidney tumours is more pronounced in the female rats (Fig. 2), where a higher tumour yield is present in all DMN plus EMS treated groups than in males (Fig. 3) where this effect is confined to the group receiving the highest dose of EMS combined with DMN. The higher multiplicity of tumours as well as the number of lung tumours in rats receiving the combined treatment (Table I and II) suggest that the carcinogenic additive effect is not confined to the kidney but is present to a lesser extent in other organs as well.

The induction of kidney tumours in rats treated with a single dose of various alkylating agents has already been exploited to correlate quantitatively the levels of alkylation in this organ and the tumour yield. Swann and Magee (1968, 1971) examined the amount of alkylation in kidney nucleic acids by dimethylnitrosamine, N-nitroso-N-methylurea, methylmethanesulphonate and the corresponding ethyl derivatives and they found a lack of correlation between the amount of alkylation of N-7 in guanine residues by each compound and their carcinogenic activity. In particular, the extent of conversion of guanine to 7-methylguanine was closely similar in the kidneys with dimethylnitrosamine and nitrosomethylurea, both of which induce tumours in this organ. With methylmethanesulphonate, however, the yield of 7-methylguanine in the kidney nucleic acids was of the same order as that found with the 2 nitroso compounds, but no kidney tumours were observed in the rats surviving a single dose of this compound. Similar results were obtained with the corresponding ethylating agents, among which a single dose of 270 mg/kg of EMS produced 5–10 times more 7-ethylguanine in rat kidney DNA than a carcinogenic dose of diethylnitrosamine or ethylnitrosourea. In these studies, a dose as large as 350 mg/kg body weight of EMS failed to induce kidney tumours; in the present studies, however, a single dose of 100 mg/kg body weight was sufficient to induce a low incidence of kidney tumours. However, these experiments (Swann and Magee, 1968, 1971) compared only the extent of alkylation of N-7 of guanine residues, which is the major alkylation site in nucleic acid bases, by these compounds. The question whether other alkylated sites in nucleic acids may be more important in the initiation of the carcinogenic process has been reviewed by Lawley (1972). As originally suggested by Loveless (1969) and substantiated by Gerchman and Ludlum (1973), alkylation of the 0–6 atom of guanine can cause mispairing of bases and thus induce transition mutations. O'Connor, Capps and Craig (1973) have shown that 0–6-methylguanine is present in hepatic DNA from rats treated with DMN, but none was detected after treatment of the animals with methylmethanesulphonate. EMS is known to react *in vitro* to the 0–6 position of guanine residues of DNA (Lawley, 1972).

The additive effect in the induction of kidney tumours in rats by a single dose of DMN and EMS, demonstrated in these studies, suggests that it might be the result of the total nucleic acid alkylation by these two compounds. However, which specific site(s) of alkylated nucleic acid bases is (are) involved in initiating the carcinogenic process remains to be established. Another factor, that could be related to the present findings is the impairment by the EMS of the cellular immune response which is known to occur with various alkylating agents (Krüger, 1972).

We wish to thank Dr N. Breslow and Dr N. Day for the statistical evaluation of the results and Miss Amanda Pickett for her secretarial assistance.

REFERENCES

Alexander, P. & Connell, D. I. (1963) In *Cellular Basis and Aetiology of Late Somatic Effects of Ionising Radiation.* Ed. R. J. C. Harris. London: Academic Press.

GERCHMAN, L. L. & LUDLUM, D. B. (1973) The Properties of 0-6-Methylguanine in Template for RNA Polymerase. *Biochim. biophys. Acta*, **308**, 310.

HARD, G. C. & BUTLER, W. H. (1970) Cellular Analysis of Renal Neoplasia: Induction of Renal Tumours in Dietary-conditioned Rats by Dimethylnitrosamine with a Reappraisal of Morphological Characteristics. *Cancer Res.*, **30**, 2796.

HRUSHESKY, W., SAMPSON, D. & MURPHY, G. P. (1972) Carcinogenicity of Ethylmethanesulphonate. *J. natn. Cancer Inst.*, **49**, 1077.

KAPLAN, E. L. & MEIER, P. (1958) Non-parametric Estimation from Incomplete Observation. *J. Am. statist. Ass.*, **53**, 457.

KRÜGER, G. R. F. (1972) Morphology of Chemical Immunosuppression. *Adv. Pharmacol. Chemother.*, **10**, 1.

LAWLEY, P. D. (1972) The Action of Alkylating Mutagens and Carcinogens on Nucleic Acids: N-Methyl-N-nitroso Compounds as Methylating Agents. In *Topics in Chemical Carcinogenesis*. Ed. W. Nakahara, University of Tokyo Press.

LOVELESS, A. (1969) Possible Relevance of 0–6 Alkylation of Deoxyguanosine to the Mutagenicity and Carcinogenicity of Nitrosamines and Nitrosamides. *Nature, Lond.*, **223**, 206.

MAGEE, P. N. & BARNES, J. M. (1959) The Experimental Production of Tumours in the Rat by Dimethylnitrosamine (N-Nitroso-Dimethylamine), *Acta Un. int. Cancr.*, **15**, 187.

MAGEE, P. N. & BARNES, J. M. (1962) Induction of Kidney Tumours in the Rat with Dimethylnitrosamine (N-Nitroso Dimethylamine). *J. Path. Bact.*, **84**, 19.

MANTEL, N. (1966) Evaluation of Survival Data and Two New Rank Order Statistics Arising in its Consideration. *Cancer chemother. Rep.*, **50**, 163.

McLEAN, A. E. M. & MAGEE, P. N. (1970) Increases in Renal Carcinogenesis by Dimethylnitrosamine in Protein Deficient Rats. *Br. J. exp. Path.*, **51**, 587.

O'CONNOR, P. J., CAPPS, M. J. & CRAIG, A. W. (1973) Comparative Studies of the Hepatocarcinogen N,N-Dimethylnitrosamine *in vivo*: Reaction Sites in Rat Liver DNA and the Significance of their Relative Stabilities. *Br. J. Cancer*, **27**, 153.

RIOPELLE, J. L. & JASMIN, G. (1969) Nature, Classification and Nomenclature of Kidney Tumours Induced in the Rat by Dimethylnitrosamine. *J. natn. Cancer Inst.*, **42**, 643.

SCHMIDT, J. D. & MURPHY, G. P. (1966) Urinary Lactic Dehydrogenase Activity in Rats with Dimethylnitrosamine Induced Renal Tumours. *Invest. Urol.*, **4**, 57.

SWANN, P. F. & MAGEE, P. N. (1968) The Alkylation of Nucleic Acids of the Rat by N-methyl-N-Nitrosourea, Dimethylnitrosamine, Dimethylsulphate and Methyl Methanesulphonate. *Biochem. J.*, **110**, 39.

SWANN, P. F. & MAGEE, P. N. (1969) Induction of Rat Kidney Tumours by Ethyl Methanesulphonate and Nervous Tissue Tumours by Methyl Methanesulphonate and Ethyl Methanesulphonate. *Nature, Lond.*, **223**, 947.

SWANN, P. F. & MAGEE, P. N. (1971) The Alkylation of N-7 of Guanine of Nucleic Acids of the Rat by Diethylnitrosamine, N-ethyl-N-Nitrosourea and Ethyl Methanesulphonate. *Biochem. J.*, **125**, 841.


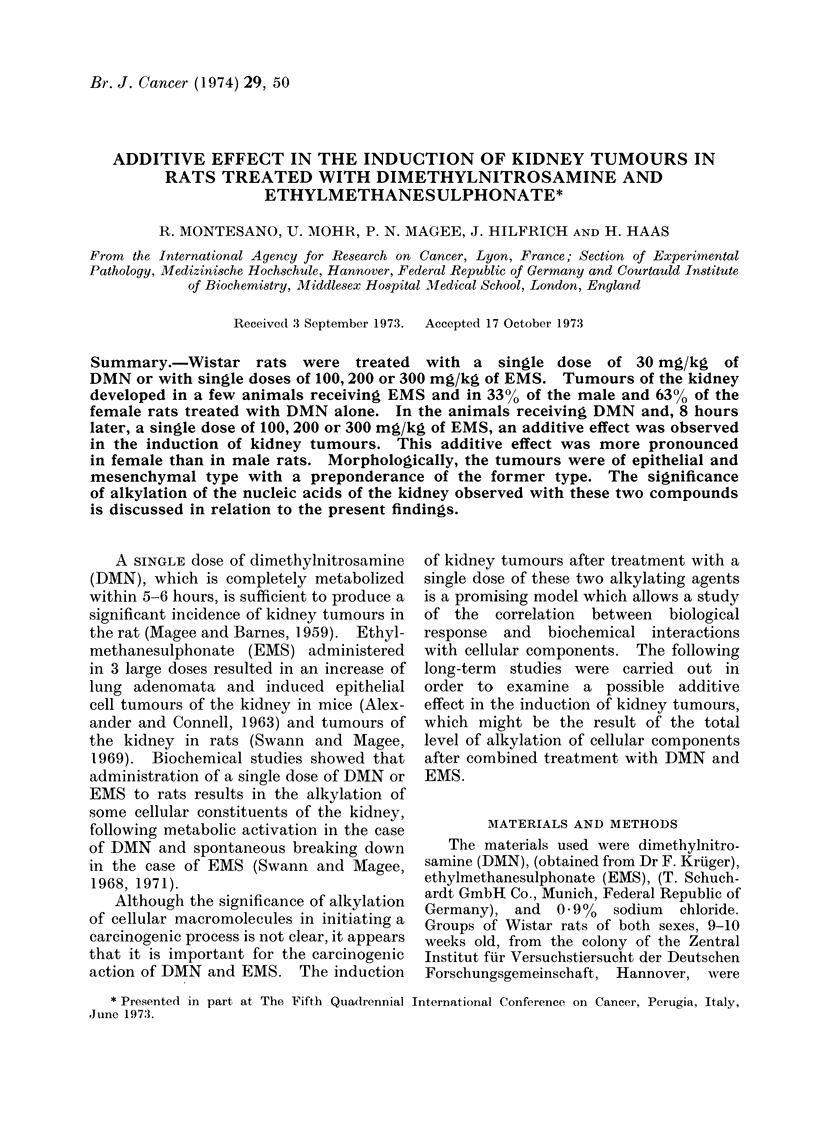

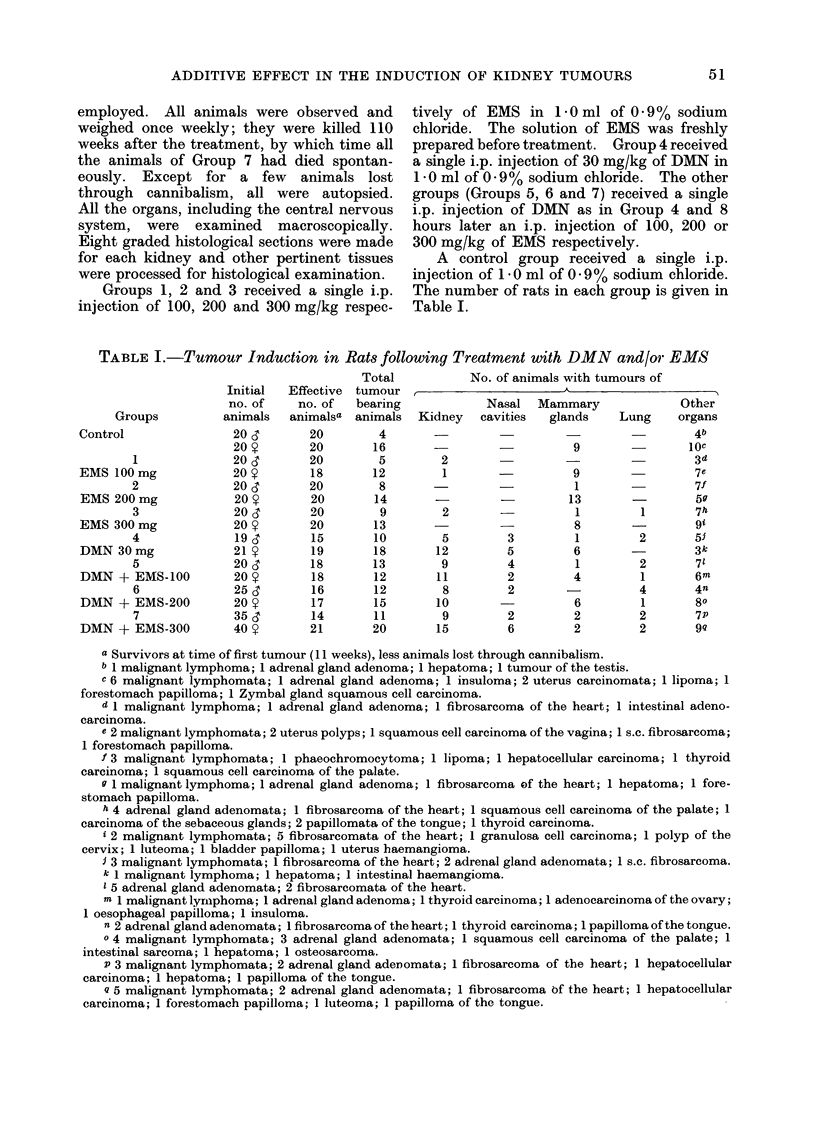

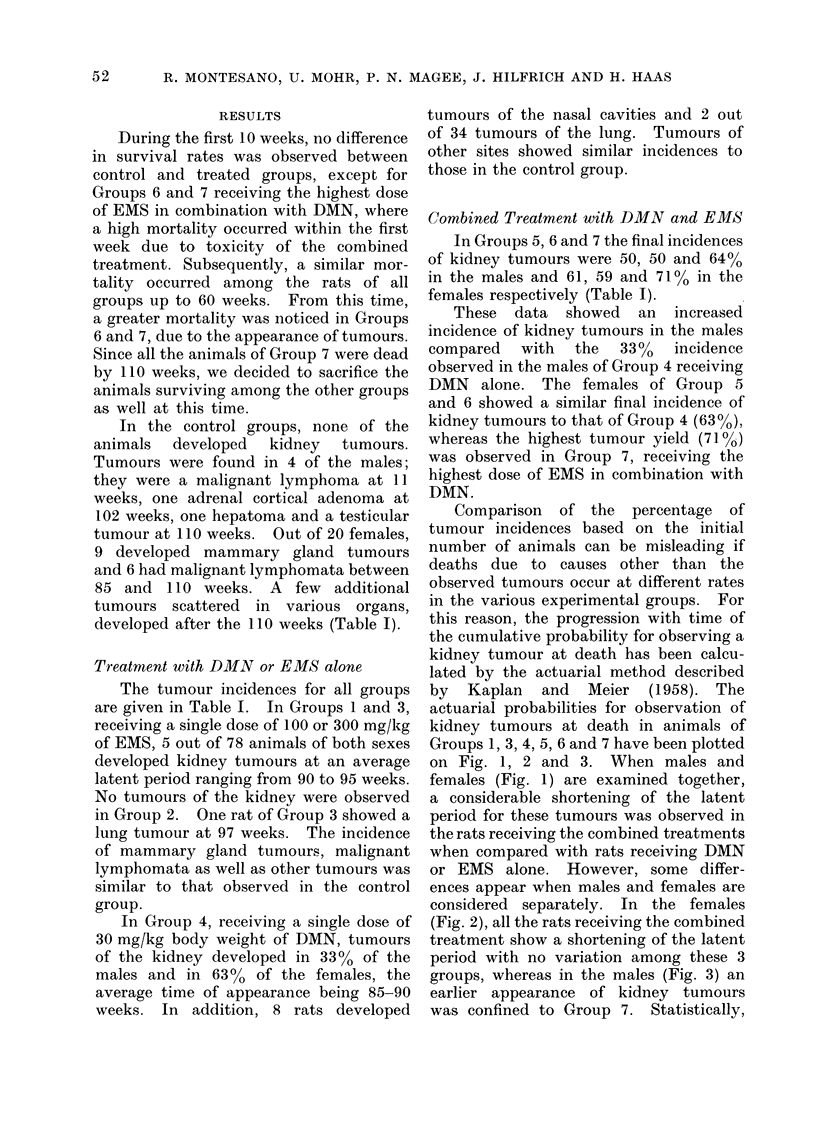

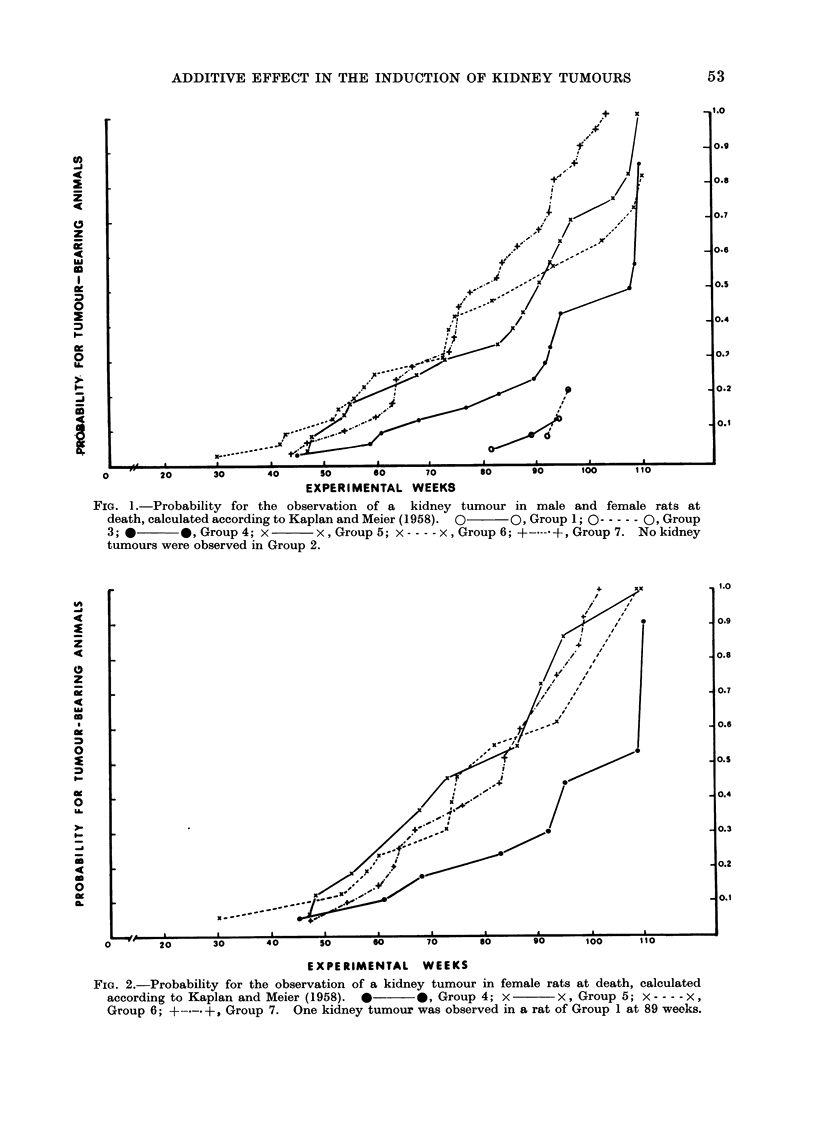

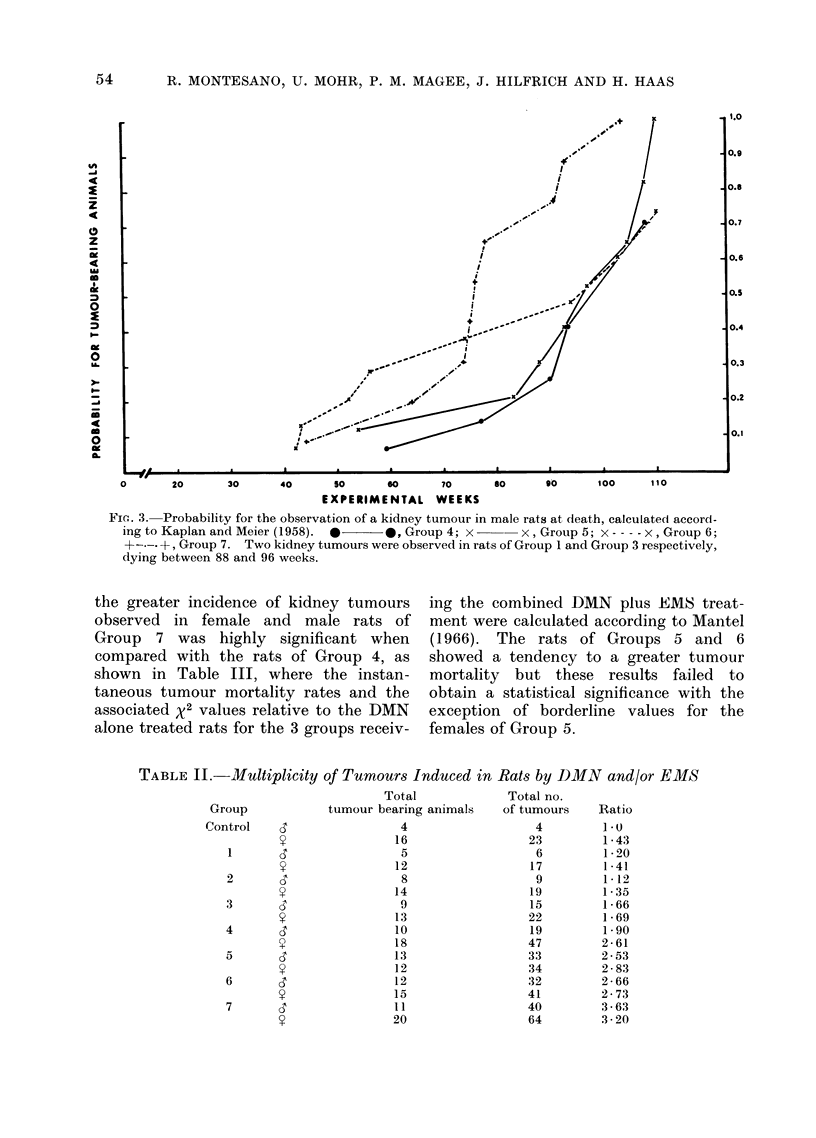

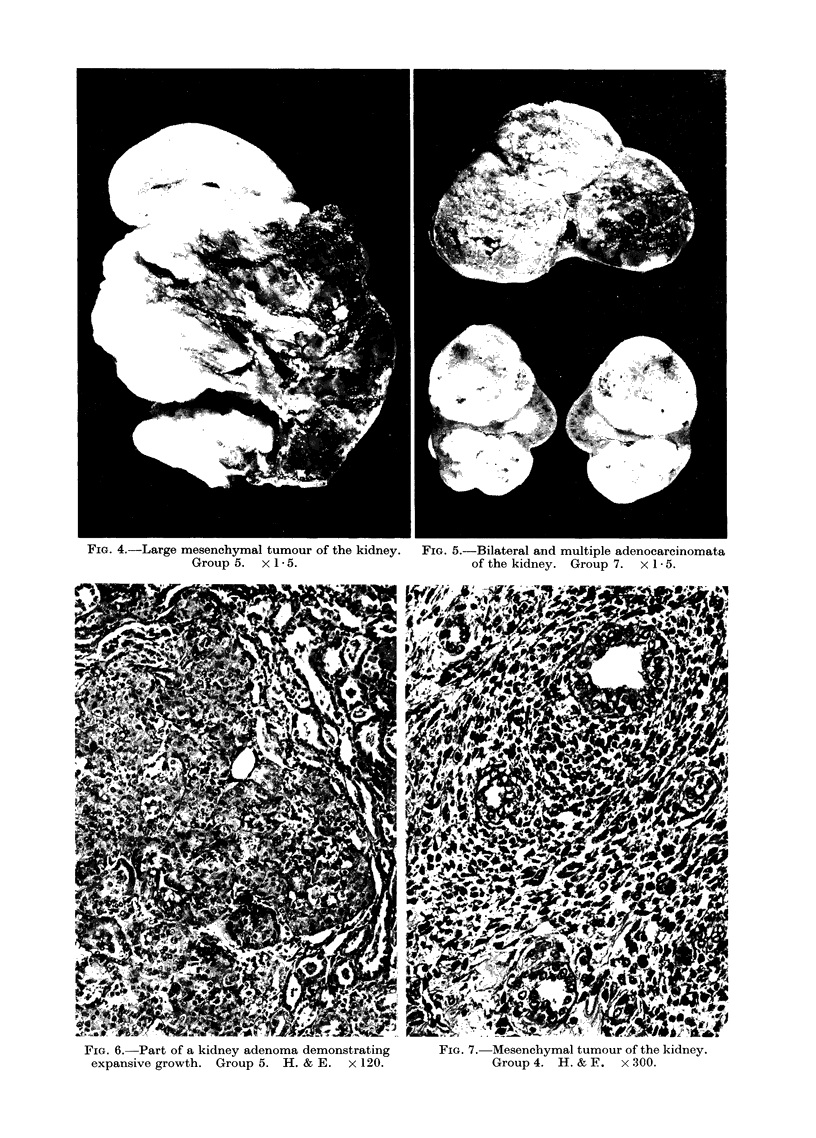

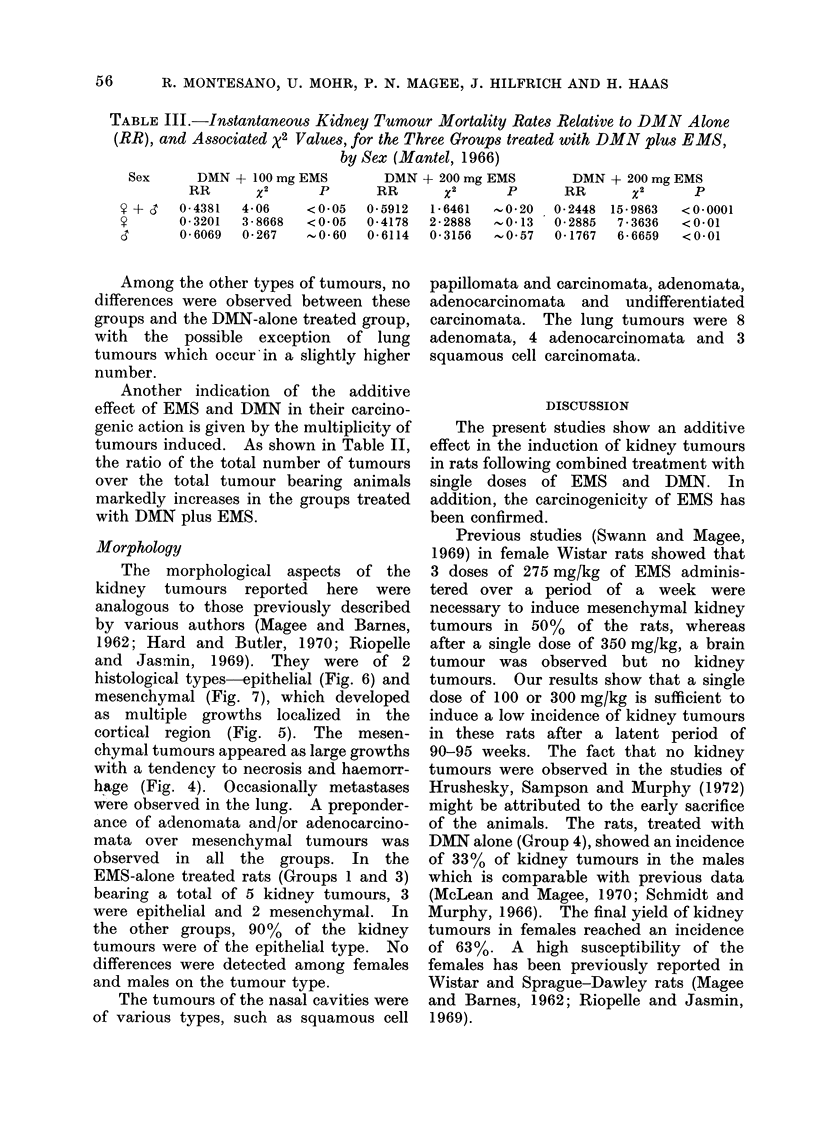

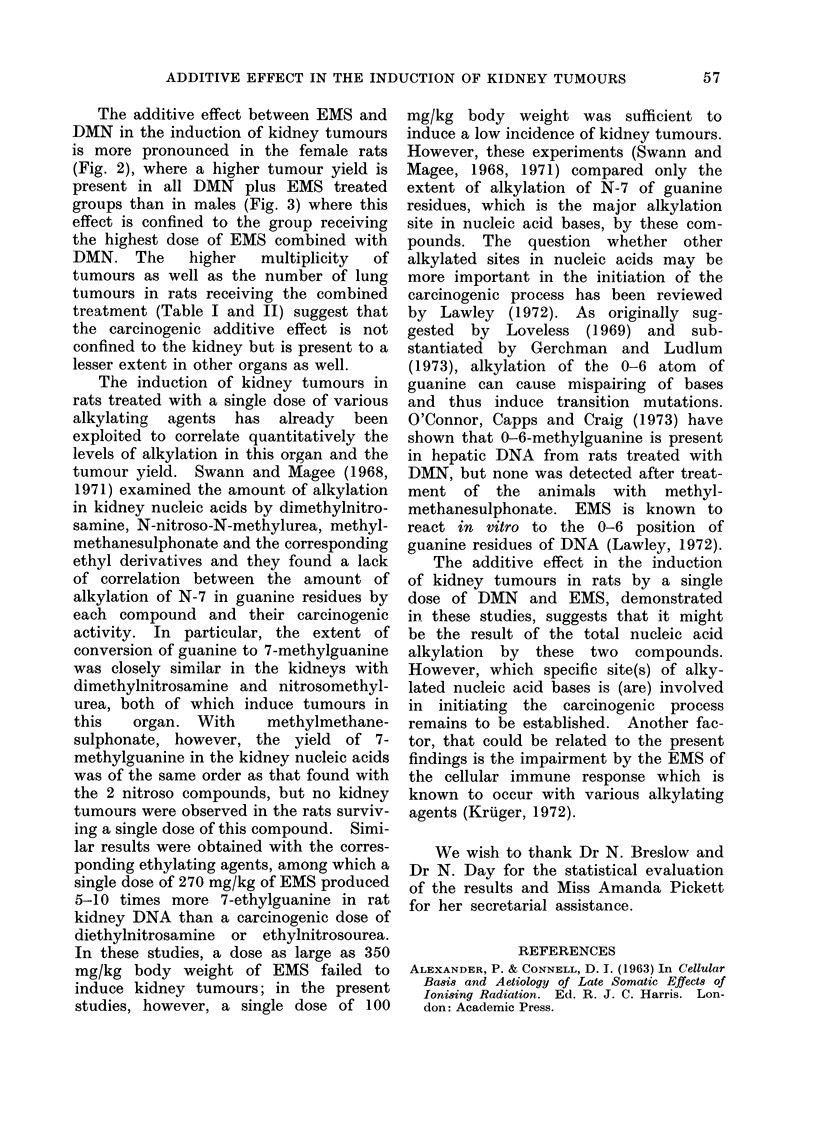

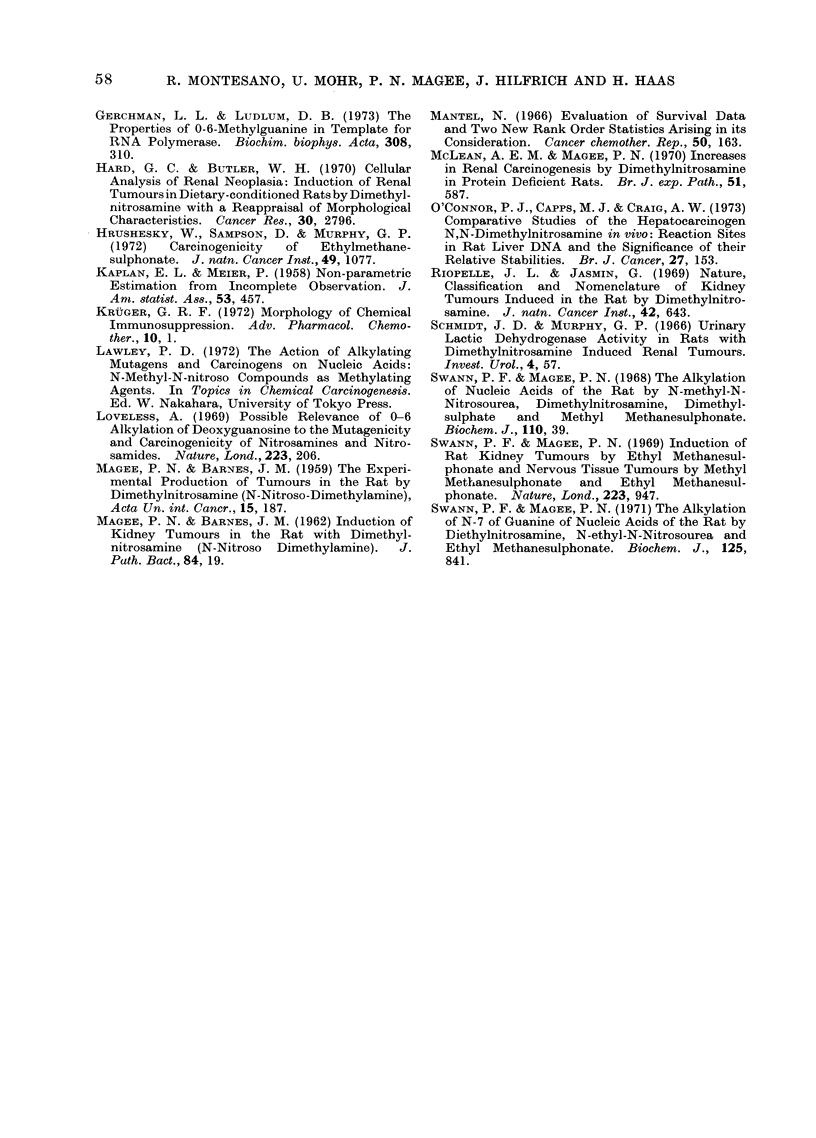


## References

[OCR_00881] Gerchman L. L., Ludlum D. B. (1973). The properties of O 6 -methylguanine in templates for RNA polymerase.. Biochim Biophys Acta.

[OCR_00887] Hard G. C., Butler W. H. (1970). Cellular analysis of renal neoplasia: induction of renal tumors in dietary-conditioned rats by dimethylnitrosamine, with a reappraisal of morphological characteristics.. Cancer Res.

[OCR_00894] Hrushesky W., Sampson D., Murphy G. P. (1972). Carcinogenicity of ethylmethanesulfonate.. J Natl Cancer Inst.

[OCR_00904] Krueger G. R. (1972). Morphology of chemical immunosuppression.. Adv Pharmacol Chemother.

[OCR_00916] Loveless A. (1969). Possible relevance of O-6 alkylation of deoxyguanosine to the mutagenicity and carcinogenicity of nitrosamines and nitrosamides.. Nature.

[OCR_00928] MAGEE P. N., BARNES J. M. (1962). Induction of kidney tumours in the rat with dimethylnitrosamine (N-nitrosodimethylamine).. J Pathol Bacteriol.

[OCR_00922] MAGEE P. N., BARNES J. M. (1959). The experimental production of tumours in the rat by dimethylnitrosamine (N-nitroso dimethylamine).. Acta Unio Int Contra Cancrum.

[OCR_00934] Mantel N. (1966). Evaluation of survival data and two new rank order statistics arising in its consideration.. Cancer Chemother Rep.

[OCR_00939] McLean A. E., Magee P. N. (1970). Increased renal carcinogenesis by dimethyl nitrosamine in protein deficient rats.. Br J Exp Pathol.

[OCR_00945] O'Connor P. J., Capps M. J., Craig A. W. (1973). Comparative studies of the hepatocarcinogen N,N-dimethylnitrosamine in vivo: reaction sites in rat liver DNA and the significance of their relative stabilities.. Br J Cancer.

[OCR_00952] Riopelle J. L., Jasmin G. (1969). Nature, classification, and nomenclature of kidney tumors induced in the rat by dimethylnitrosamine.. J Natl Cancer Inst.

[OCR_00958] Schmidt J. D., Murphy G. P. (1966). Urinary lactic dehydrogenase activity in rats with dimethylnitrosamine induced renal tumors.. Invest Urol.

[OCR_00971] Swann P. F., Magee P. N. (1969). Induction of rat kidney tumours by ethyl methanesulphonate and nervous tissue tumours by methyl methanesulphonate and ethyl methanesulphonate.. Nature.

[OCR_00964] Swann P. F., Magee P. N. (1968). Nitrosamine-induced carcinogenesis. The alklylation of nucleic acids of the rat by N-methyl-N-nitrosourea, dimethylnitrosamine, dimethyl sulphate and methyl methanesulphonate.. Biochem J.

[OCR_00978] Swann P. F., Magee P. N. (1971). Nitrosamine-induced carcinogenesis. The alkylation of N-7 of guanine of nucleic acids of the rat by diethylnitrosamine, N-ethyl-N-nitrosourea and ethyl methanesulphonate.. Biochem J.

